# The impact of large scale licensing examinations in highly developed countries: a systematic review

**DOI:** 10.1186/s12909-016-0729-7

**Published:** 2016-08-19

**Authors:** Julian Archer, Nick Lynn, Lee Coombes, Martin Roberts, Tom Gale, Tristan Price, Sam Regan de Bere

**Affiliations:** 1 Collaboration for the Advancement of Medical Education Research and Assessment, Plymouth University Peninsula Schools of Medicine & Dentistry, Plymouth, Devon UK; 2 Centre for Medical Education, Cardiff University School of Medicine, Heath Park, Cardiff, UK

**Keywords:** National licensing examination, Validity, Impact, Assessment, Systematic review

## Abstract

**Background:**

To investigate the existing evidence base for the validity of large-scale licensing examinations including their impact.

**Methods:**

Systematic review against a validity framework exploring: Embase (Ovid Medline); Medline (EBSCO); PubMed; Wiley Online; ScienceDirect; and PsychINFO from 2005 to April 2015. All papers were included when they discussed national or large regional (State level) examinations for clinical professionals, linked to examinations in early careers or near the point of graduation, and where success was required to subsequently be able to practice. Using a standardized data extraction form, two independent reviewers extracted study characteristics, with the rest of the team resolving any disagreement. A validity framework was used as developed by the American Educational Research Association, American Psychological Association, and National Council on Measurement in Education to evaluate each paper’s evidence to support or refute the validity of national licensing examinations.

**Results:**

24 published articles provided evidence of validity across the five domains of the validity framework. Most papers (*n* = 22) provided evidence of national licensing examinations relationships to other variables and their consequential validity. Overall there was evidence that those who do well on earlier or on subsequent examinations also do well on national testing. There is a correlation between NLE performance and some patient outcomes and rates of complaints, but no causal evidence has been established.

**Conclusions:**

The debate around licensure examinations is strong on opinion but weak on validity evidence. This is especially true of the wider claims that licensure examinations improve patient safety and practitioner competence.

## Background

Medical regulation has historically involved establishing who is appropriately qualified to call themselves a medical doctor and keeping certain people, such as “barbers” [1] and “charlatans” [2], out. But medical regulators have moved away from this static approach, of simply holding a register, to a more dynamic and prospective one.

Much of the attention has perhaps understandably focused at the beginning of clinical practice. This transition point from medical school into the workplace is also often a point at which international medical graduates enter the workforce. One way in which regulators have controlled entry into practice within their borders has been to establish national licensing examinations (NLE); the most notable of which is perhaps the United States Medical Licensing Examination (USMLE) from the National Medical Board of Examiners (NBME) [3].

It is easy to understand why the concept of a licensing exam is hailed as important. They sit at the point at which medical schools graduate their students. Only those who achieve the required standards are then allowed to practice in the jurisdiction. In this way, the advocators argue, a nation’s population is reassured that only capable doctors who can practice safely are qualified. Moreover, the centrality of NLEs to the continuing debates within medical education concerning assessment is brought into sharper focus by the predictions of their inevitable proliferation. Swanson and Roberts have recently opined on this subject, arguing that, amongst other factors, the increase in the numbers and diversity of medical schools and the increasing mobility of the medical workforce will increase the demand for NLEs globally [4]. But exactly what form NLEs should take, what they should cover and who they should assess remains a source of debate [5–11], as doctors and other healthcare workers increasingly wish to move across national or regional (state) boundaries [5, 12–16].

The United Kingdom (UK) does not currently have a NLE but has historically relied on external examiners – visiting medical educators from other organizations – and General Medical Council (GMC) inspections to assure quality across UK medical schools. Doctors from overseas take a different route into licensure in the UK; predominately through the Professional and Linguistic Assessments Board (PLAB) examination [17].

However at the end of 2014 the GMC announced that it planned to establish a NLE and in June 2015 it laid out a timeframe for the introduction of a ‘Medical Licensing Assessment’ (MLA) which will ultimately be taken by all UK graduates and non-European Economic Area (EEA) graduates who wish to practice in the UK by 2021 [18]. As European Law stands EEA graduates will be exempt under freedom of movement legislation [19].

As part of the process of developing this new NLE, the GMC commissioned researchers at the Plymouth University Peninsula School of Medicine to undertake a systematic review of the international literature to establish the evidence base for the validity including the impact of NLEs and to identify best practice [20]. The findings are relevant not just to the UK, but to regulators and policy makers in comparable countries who are considering introducing or reforming a national licensing system. In particular, by examining the existing evidence for both the positive and negative consequences of national licensing, we contribute to a wider and more informed evidence base upon which to base regulatory decisions.

## Methods

### Data sources and searches

We conducted a systematic review following guidance on the conduct of narrative synthesis in systematic reviews [21].

We carried out a systematic electronic search in Embase (Ovid Medline), Medline (EBSCO), PubMed, Wiley Online, ScienceDirect, and PsychINFO. These databases varied in relation to both subject content and size. In those databases with medical or healthcare profession sections, such as EBSCO and EMBASE, searches were only conducted within these areas.

All databases were searched from 2005 to April 2015. We used a combination of relevant keywords to construct the search strategy including “national licensing examination”, “doctor”, “dentist”, “nurse”, “midwife”, “healthcare professional”, “international medical graduate”, “accreditation”, “credentialing”, “registration”, and “certification”.

One author (NL) conducted the first screening of potentially relevant records based on titles and abstract, and four authors (NL, JA, MR and LC) independently performed the final selection of included papers based on full text evaluation. Consensus between the reviewers was used to resolve any disagreement supported by all the authors. A review of websites of medical regulators or those bodies with responsibility for licensing doctors and healthcare professionals in 49 ‘very high human development’ countries similar to the UK was also undertaken [22]. The United Nations Development Programme (UNDP) measures ‘Human Development’ by evaluating and assessing an index of component parts. These are: life expectancy at birth, mean years of schooling, expected years of schooling, gross national income (GNI) per capita. From this, countries are then ranked as having ‘very high’, to ‘low’ human development - those countries in the ‘very high’ category include the UK [22].

Secondly, and in addition to the search process, we contacted, via the GMC and the International Association of Medical Regulatory Authorities (IAMRA), medical regulators and licensing authorities in the 49 countries. We sought to gather information (grey or unpublished) which had shaped the thinking of regulators when planning, or excluding, licensure examinations.

Finally, we reviewed the websites of each of the bodies involved in medical regulation in the 49 countries with the intention of identifying any source of underlying validity evidence, such as references to papers, assessment manuals etc., for NLEs where we found them.

### Study selection

From the published literature, we included all papers that discussed national or large regional (State level) examinations for medical and healthcare professionals published since 2005. Papers were eligible if they were linked to examinations that were normally taken in early careers or near the point of graduation, and for which success in the examination was required to enter practice. There were no restrictions on language but only examinations in countries “comparable to the UK” were included [22]. Specialist examinations were excluded, as were exams at local or institutional level. We only included papers that discussed empirical evidence for the validity of NLEs. We have discussed commentaries, such as editorial and opinion pieces, elsewhere [23].

### Data extraction, synthesis and analysis

Using a standardized data extraction form, three pairs of reviewers (NL with one of JA, MR and LC) independently extracted study characteristics from the included papers, with the rest of the team involved to resolve any disagreement. We recorded study characteristics for each paper’s evidence to support or refute the validity of NLEs. We used a validity framework developed by the American Educational Research Association (AERA), the American Psychological Association (APA), and the National Council on Measurement in Education (NCME), as described in Downing paper for medical education in 2003 [24]. The APA framework, which has been described as “the current standard of assessment validation” [25], is rooted in the assertion that assessments themselves are not valid or invalid, but may offer “more or less evidence” for a specific interpretation of assessment data at a “given point in time and only for some well-defined population” [24]. This reorientation of focus is particularly suited to the assessment of NLEs given that the different types of exam operate within different systems and in different contexts. Furthermore, with its five domains of validity evidence, the APA framework allows us to examine not only the evidence that NLEs measure what they intend to measure, but also, with its inclusion of the *consequences* domain, we can focus on the “impact, beneficial or harmful and intended or unintended, of the [national licensing] assessment” [26].

Importantly, such evidence can be differentiated from correlation evidence showing a relationship between test scores and other criteria related to improved performance. This understanding of impact as consequences allowed us to differentiate between evidence showing a correlation between NLE test results and doctor performance, from evidence for improved performance as a consequence of introducing NLEs. This also allowed us to expand the focus on the consequence of NLEs, to include both intended and unintended effects and its impact on different groups, such as course designers, regulators, policy-makers, as well as the doctors and most importantly patients.

We extracted the evidence and used this framework to systematically organize the evidence found in the literature review and to structure our analysis and reporting. The framework categorizes validity into five sources of evidence; content, response process, internal structure, relationship to other variables and consequences. More details, with examples, can be seen in Table 1. Each reviewer assessed each paper for the quality of evidence mapped to the framework including limitation of study design, inconsistency of results, imprecision, and publication bias.

**Table 1:** Summary of the validity framework adopted from Downing [24]

Type of validity evidence (and description)	Examples of validity evidence found in medical education
Content * Content validity includes the outline and plan for the test. The principal question to ask is whether the content of the test is sufficiently similar to and representative of the activity or performance it is intended to measure?*	The outlines, subject matter domains, and plan for the test as described in the test ‘blueprint.’ Mapping the test content to curriculum specifications and defined learning outcomes. The quality of the test questions and the methods of development and review used to ensure quality. Expert input and judgements and how these are used to judge the representativeness of the content against the performance it is intended to measure.
Response Process * Response process is concerned with how all the participants - candidates and officials - respond to the assessment. It is part of the quality control process.*	The clarity of the pre-test information given to candidates. The processes of test administration, scoring, and quality control. The guidelines for scoring and administration. The performance of judges and observers. Quality control and accuracy of final marks, scores, and grades.
Internal Structure * Is the assessment structured in such a way as to make it reliable, reproducible, and generalizable? Are there any aspects of the assessment’s structure that might induce bias?*	The statistical or psychometric characteristics of the test such as: • Item performance e.g. difficulty • Factor structure and internal consistency of subscales • Relationship between different parts of the test • Overall reliability and generalizability Matters relating to bias and fairness.
Relationship to other variables * The relationship to other variables is concerned with the connection between test scores and external variables.*	The correlation or relationship of test scores to external variables such as: • Scores in similar assessments with which we might expect to find strong positive correlation. • Scores in related but dissimilar assessments e.g. a knowledge test and an Objective Structured Clinical Examination (OSCE), where weaker correlations might be expected. Generalizability of evidence and limitations such as study design, range restriction, and sample bias.
Consequences * Consequences or evidence of impact is concerned with the intended or unintended consequences assessment may have on participants or wider society. It may include whether assessments provide tangible benefits or whether they have an adverse or undesirable impact.*	The intended or unintended consequences of the assessment on participants (such as failure) or wider societal impacts. This includes the wider impact of the assessment when viewed as an intervention. The methods used to establish pass/fail scores. False positives and false negatives.

## Results

Our initial search yielded 202 documents suitable for more detailed review. These records consisted of qualitative, quantitative, and mixed methodology studies, as well as editorials, opinion pieces and (mostly expert) personal views from across the healthcare professions. In our review of the national regulatory websites we also uncovered some relevant blogs and other social media offering anecdotes and advice. Overall, the internet searches yielded only 14 potentially relevant documents of the overall total. We received 11 replies from our approach to international medical regulatory authorities. These contained 3 references to literature that we had already obtained through our searches but no other additional manuals, documents or information sources.

As a team, we screened the abstracts for relevance and reduced the total number to 104 records. On full text evaluation that followed, 30 papers were excluded for not meeting the inclusion criteria. The total number of papers included in the final review prior to the framework mapping exercise was therefore 73 papers.

**Fig. 1:**
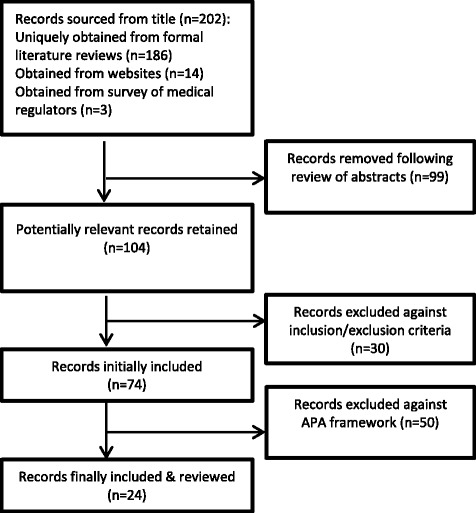
Overview of the literature search process

We found a lot of debate in the literature but much less evidence. After we mapped the papers to the validity framework, only 24 of the 73 papers were found to contain validity evidence for licensing examinations. The remaining 50 papers consisted of informed opinion, editorials, or simply described and contributed to the continuing debate. We summarize the overall review process in Fig. 1.

Table 2 summarizes the 24 papers mapped to the validity framework. Of these reviewed papers only four offered evidence for content validity [27–30], three for response process [27, 29, 31], and four for internal structure [27–29, 32].

**Table 2:** Papers providing empirical evidence for the validity of licensing examinations mapped against sources of –validity evidence [24]

Content	Response process	Internal structure	Relationship to other variables	Consequences
CEUP (2008): ‘Comprehensive Review of USMLE Summary of the Final Report and Recommendations’ Lillis, S., Stuart, M., Sidonie, Takai, N. (2012): ‘New Zealand Registration Examination (NZREX Clinical): 6 years of experience as an Objective Structured Clinical Examination (OSCE)’ Ranney, R.R. (2006): ‘What the Available Evidence on Clinical Licensure Exams Shows.’ Guttormsen, S., Beyeler, C., Bonvin, R., Feller, S., Schirlo, C., Schnabel, K., Schurter, T., Berendonk, C. (2013): ‘The new licensing examination for human medicine: from concept to implementation.’	Lillis, S., Stuart, M., Sidonie, Takai, N. (2012): ‘New Zealand Registration Examination (NZREX Clinical): 6 years of experience as an Objective Structured Clinical Examination (OSCE)’ Seyfarth et al., (2010): ‘Grades on the Second Medical Licensing Examination in Germany Before and After the Licensing Reform of 2002.’ Guttormsen, S., Beyeler, C., Bonvin, R., Feller, S., Schirlo, C., Schnabel, K., Schurter, T., Berendonk, C. (2013): ‘The new licensing examination for human medicine: from concept to implementation.’	Harik, P., Clauser, B.E., Grabovsky, I., Margolis, M.J., Dillion, G.F., Boulet, J.(2006): ‘Relationships among subcomponents of the USMLE Step 2 Clinical Skills examination, the Step 1, and the Step 2 Clinical Knowledge examinations. Lillis, S., Stuart, M., Sidonie, Takai, N. (2012): ‘New Zealand Registration Examination (NZREX Clinical): 6 years of experience as an Objective Structured Clinical Examination (OSCE)’ Ranney, R.R. (2006): ‘What the Available Evidence on Clinical Licensure Exams Shows.’ Guttormsen, S., Beyeler, C., Bonvin, R., Feller, S., Schirlo, C., Schnabel, K., Schurter, T., Berendonk, C. (2013): ‘The new licensing examination for human medicine: from concept to implementation.’	Cuddy, M.M., Dillion, G.F., Holtman, M.C., Clauser, B. (2006): ‘A Multilevel Analysis of the Relationships Between Selected Examinee Characteristics and United States Medical Licensing Examination Step 2 Clinical Knowledge Performance: Revisiting Old Findings and Asking New Questions.’ Harik, P., Clauser, B.E., Grabovsky, I., Margolis, M.J., Dillion, G.F., Boulet, J.(2006): ‘Relationships among subcomponents of the USMLE Step 2 Clinical Skills examination, the Step 1, and the Step 2 Clinical Knowledge examinations.’ Guttormsen, S., Beyeler, C., Bonvin, R., Feller, S., Schirlo, C., Schnabel, K., Schurter, T., Berendonk, C. (2013): ‘The new licensing examination for human medicine: from concept to implementation.’ Hecker K, & Violato, C. (2008): ‘How much do differences in Medical Schools Influence Student Performance? A Longitudinal Study Employing Hierarchical Linear Modelling. Kenny, S., McInnes, M., Singh, V. (2013): ‘Associations between residency selection strategies and doctor performance: a meta-analysis.’ McManus, I., & Wakeford, R. (2014): ‘PLAB and UK graduates performance on MRCP(UK) and MRCGP examinations: data linkage study.’ Norcini et al., (2014): ‘The relationship between licensing examination performance and the outcomes of care by international medical school graduates.’ Ranney, R.R. (2006): ‘What the Available Evidence on Clinical Licensure Exams Shows.’ Stewart, et al., (2005): ‘Relationship Between Performance in Dental School and Performance on a Dental Licensure Examination: An Eight Year Study.’ Sutherland, K., & Leatherman, S. (2006): ‘Regulation and Quality Improvement A Review of the Evidence.’ Tamblyn et el., (2007): ‘Physician Scores on a National Clinical Skills Examination as Predictors of Complaints to Medical Regulatory Authorities. Tiffin et al., (2014): ‘Annual Review of Competence Progression ARCP Performance of doctors who passed Professional and Linguistic Assessments Board (PLAB) tests compared with UK graduates.’ Zahn et al., (2012): ‘Correlation of National Board of Medical Examiner’s Scores with the USMLE Step 1 and Step 2 Scores.’	Ahn, D., & Ahn, S. (2007): Reconsidering the Cut Score of the Korean National Medical Licensing Examination Gilliland WR, Rochelle JL, Hawkins R, Dillon GF, Mechaber AJ, Dyrbye L, et al. (2008) ‘Changes in clinical skills education resulting from the introduction of the USMLE™ step 2 clinical skills (CS) examination.’ Green, M., Jones, P., Thomas Jr, J.X. (2009): ‘Selection Criteria for Residency: Results of a National Program Directors Survey.’ Guttormsen, S., Beyeler, C., Bonvin, R., Feller, S., Schirlo, C., Schnabel, K., Schurter, T., Berendonk, C. (2013): ‘The new licensing examination for human medicine: from concept to implementation.’ Holtzman et al., (2014): ‘International variation in performance by clinical discipline and task on the United States Medical Licensing Examination Step 2 Clinical Knowledge Component.’ Kenny, S., McInnes, M., Singh, V. (2013): ‘Associations between residency selection strategies and doctor performance: a meta-analysis.’ Kugler, A. D, & Sauer, R.M. (2005): Doctors without Borders? Relicensing Requirements and Negative Selection in the Market for Physicians.’ Lillis, S., Stuart, M., Sidonie, Takai, N. (2012): ‘New Zealand Registration Examination (NZREX Clinical): 6 years of experience as an Objective Structured Clinical Examination (OSCE)’ Margolis et al., (2010): ‘Validity Evidence for USMLE Examination Cut Scores: Results of a Large Scale Survey’ Musoke, S. (2012): ‘Foreign Doctors and the Road to a Swedish Medical License.’ Ranney, R.R. (2006): ‘What the Available Evidence on Clinical Licensure Exams Shows.’ Stewart, et al., (2005): ‘Relationship Between Performance in Dental School and Performance on a Dental Licensure Examination: An Eight Year Study.’ ’ Wenghofer et al., (2009): ‘Doctors’ Scores on National Qualifying Examinations Predict Quality of Care in Future Practice.’

The majority of the literature focused on the relationship of licensing examinations to other variables and on consequential validity evidence. A comparison of licensing examinations to other assessment methods and exploration of their impact provides an opportunity to explore whether they provide unique or compelling validity evidence to the regulatory/safety debate, over and above other forms of assessment. Arguably, this after all should inform the basis for their implementation.

### Relationship to other variables as evidence for validity

The papers that explored the relationship to other variables, as evidence for validity, we sub-grouped into three areas of enquiry: prior and future performance by individuals in examinations; relationship to patient outcomes and complaints; and specifically the variation in performance between home-trained doctors and IMGs.

First, several authors explored the relationship between medical school examination performance and subsequent established large scale testing e.g. the USMLE [28, 33–35]. Overall they found, perhaps not surprisingly, that those who do well in medical school examinations also do well in subsequent testing. However not all the difference in performance between students can be explained by previous differences in examination results; implying that the education at different medical schools might play some role in subsequent performance and it is not simply that the best students continue to excel [33, 36].

Second, there is mixed evidence on the relationship with other variables when NLE test scores are compared with criterion based outcomes around complaints and patient welfare. Sutherland & Leatherman concluded in a 2006 review that “*there is little evidence available about [national licensing examinations’] impact on quality of care”* across the international healthcare system [37]. Since then researchers have tried to establish such a link. Both Norcini et al. (2014) and Tamblyn et al. (2007) found correlations between licensing examination performance (in the USA and Canada respectively) and subsequent specific patient outcomes [38], or rates of complaints [39]. However, as discussed in more detail below, this evidence for a correlation with other variables is not supported by evidence for better patient outcomes as a consequence of NLEs.

Third, a series of papers each demonstrated that IMGs do less well in large scale testing [32, 35, 40, 41]. In two UK studies, IMGs who were licensed through the PLAB test performed less well than home graduates at specialty examination level [41], and at in-training reviews; [35] and in each case these correlated with their prior performance on the PLAB. In both cases the authors argue that standards should be raised by elevating the PLAB cut score or introducing different assessment methods. Though a NLE would arguably provide a direct comparison between all working doctors, the authors also highlight the possibility that raising standards might lead to some IMGs not wishing or being able to work in the UK leading to workforce shortages [35, 41]. Some argue that the differences were due to a lack of proficiency in spoken English [32, 40], but a paper from Switzerland found that while IMGs did less well than Swiss candidates in their Federal Licensing Examination, the IMGs’ lower scores were in areas other than communication skills [27].

### Consequential validity

An important finding of this review is the lack of evidence that patient outcomes improve as a consequence of the introduction of national licensing exams. As noted above, there is evidence that performance in NLEs is correlated to improved patient outcomes and less complaints, but this has not been shown to be as a consequence of national licensing exams.

Although the aforementioned studies by Norcini et al. [38] and Tamblyn et al. [39] demonstrate excellent arguments for the importance of testing, and medical education more generally, their findings are limited to establishing correlations between testing and outcomes and not causation. In other words, there is evidence that better doctors do better in NLEs, but not that doctors improve as a *consequence* of introducing NLEs; this kind of before and after evidence is absent in the extant literature. One confounding factor to a causal link between testing performance and subsequent care is the fact that those who do well in the USMLE get the better jobs in the better institutions [36, 42]. These institutions are likely to play as significant a role in patient outcomes as the individual doctors they employ.

Overall, some authors argue that NLEs are not real barriers to entry into the profession, and therefore do not protect the public. For example nearly everyone who takes the USMLE passes it in the end [43]. Others raise the potential issue that IMGs are being stigmatized and disadvantaged as they try to negotiate a confusing bureaucratic process [44], and that any economic argument does not stand up as doctors always find ways to work around the system [45]. An example of this for the MLA in the UK might be IMGs seeking citizenship in an EEA partner country and then entering the UK thereby bypassing the new requirements.

There is no clear picture from the literature as to the impact of NLEs on the medical school curricula. One study on the introduction of a Step 2 Clinical Skills component of the USMLE surveyed medical educators in medical schools in the US. The study found that over one third of respondents reported changes to the “objectives, content, and/or emphasis of their curriculum” (p.325) [46]. While the study focuses only on the introduction of one new component of a licensure exam, within an already well established NLE, it does raise the question of whether NLEs can be used to focus medical schools’ attention to nationally identified skills/knowledge shortages, as appears to be the case with the clinical skills component in this study [46].

At the same time however, this raises the question that NLE exams may encourage homogeneity or a lack of innovation in curriculum design. Yet aside from one dental example in Florida [34], there appears to be no empirical evidence that NLEs encourage homogeneity or a lack of innovation and diversity – i.e. by orientating curricula to the NLE exam – in prior education programs.

## Discussions

The literature we reviewed is characterized by diversity in terms of its methodological approach, the type of evidence it provides and its overall quality and utility. There is no doubt that there is now a high degree of sophistication in the testing and assessment involved in licensure exams, particularly the USMLE. For this reason perhaps, the technical aspects of NLEs are well evidenced in the literature [30, 47, 48], assuring the examination from pedagogic and legal standpoints. However, claims made that NLEs lead to improved patient safety [8, 38, 49], enhanced quality of care [50], and the identification of doctors likely to subsequently face disciplinary action [39], are less evidenced and rely on correlations not causal evidence. While statistical correlations between NLE performance and some areas of future practice certainly exist, such an interpretation is limited by the large numbers of variables unaccounted for by this research.

For example, studies demonstrate that candidates with lower NLE scores tend to end up working in less respected institutions [36, 42] and poorer performing organizations [51]. Moreover, a comprehensive review on the role of regulation in improving healthcare by Sutherland and Leatherman [37], found “sparse” evidence to support the claims that NLE pass scores are a predictor of patient care or future disciplinary actions. Our review supports that conclusion. Swanson and Roberts appear to take the opposing view, arguing that the “evidence that better performance in NLEs is associated with better patient care seems compelling”, and that this, “aids in justifying the more widespread use of NLEs” [4]. While the literature we have reviewed here does indeed support such an “association”, this is little more than argument for assessment in general; it does not speak to the bigger, and more policy relevant, question of whether the instigation of NLEs improves performance and patient care.

That there is validity evidence for the correlation, as opposed to causation, between NLEs and doctors’ performance may in itself be an argument for national licensing [4], but this will depend on the policy purpose of the NLE. Schuwirth has recently pronounced that, “In essence the purpose of national licensing is to reassure the public that licensed doctors are safe, independent practitioners” [52]. Similarly, Swanson and Roberts point to the role of NLEs in “reassuring patients, the public, and employing organisations that, regardless of where their doctor trained, they can be sure of a minimum level of competence” [4]. However, as Schuwirth notes, public reassurance is, “at least partly, based on public perception” [52]. The danger here is a potential disjuncture between what the public, and indeed policy-makers, perceive that NLEs do, and what they actually achieve; misplaced trust in the impact of national licensing to enhance patient safety, when what they actually do is simply reassure the public, may potentially divert attention from other important aspects of medical regulation.

Lastly, there are difficult questions raised about inclusion, exclusion, and fairness in respect to IMG doctors [14]. In Sweden, which has a regulatory system similar to other countries across Europe and elsewhere, IMGs’ experiences suggest that the Swedish system may actively disadvantage competent IMG practitioners; participants viewed the Swedish system as flawed, overlong, and frustrating [44]. Such difficulties have also been highlighted by a number of Canadian studies [13, 53], providing some descriptive evidence of the way in which practitioners, provincial licensing authorities, and employers use the system to balance the demands arising from physician shortages, making it difficult for both IMGs and those that employ them to negotiate the licensing system. Meanwhile a comparative assessment of IMG policies in Australia and Canada highlighted ideological differences concerning the position of IMGs within existing medical hierarchies [14]. The Canadian approach is one of assimilation, predicated on the assumption that IMGs will seek Canadian citizenship. In contrast, Australian regulations foster a parallel but separate workforce culture more amenable to temporary licensing and with no corresponding assumptions of citizenship or even permanent residency [14]. Thus, while national licensing may provide a benchmark for physician standards, it is not clear that this will create a level playing field for IMGs. This may be further complicated by conflicts with wider systems of workforce regulation. In the EU for example, it is not clear that, given the regulations on the internal market and the free movement of labor, an individual European state would be able to enforce a NLE on a doctor that qualified within the EU [54].

### Strengths and weaknesses of the study

Our review systematically approached a large literature. Much of it was not evidence based but strong on expert opinion and debate. We used a framework to help shape and focus those papers that provided evidence of validity [24]. This framework, with its distinction between the relationship to other variables and consequences as domains of validity evidence, is particularly useful in drawing out the evidence for the discussion. We limited the studies to countries comparable with the UK using the UNDP definitions [22], but may have limited our review by only including published work from the last decade. Given that the evidence collected here relates to developed economics similar to the UK, the findings of the study are limited in their direct relevance to these categories of countries, although we hope that some of the broader issues highlighted in relation to the evidence base for NLEs will be useful to regulators and policy-makers from a wider global audience.

### Implications for clinicians and policymakers

The weakness of the evidence base exists for those who argue against national licensure examinations [7], as well as for those who advocate such a system [55–57]. While informed academics cite a variety of research studies to either rebut the pro-licensure lobby or to evaluate the relative problems and merits associated with NLEs, by their own admission there is a lack of *unequivocal* evidence and an identifiable gap in knowledge on this subject [58].

### Unanswered questions and future research

Our review suggests a significant knowledge gap exists around the impact of licensure examinations on subsequent patient care and on the profession, especially IMGs. Whilst a strong body of statistical evidence exists to show IMGs perform less well in licensure examinations than candidates from the host countries, [27, 59] the reasons for this phenomenon remain unclear. In view of the significant part IMGs play in the physician workforce of many countries *and* the apparent difficulties they present to regulators, this is an area of research that needs to be better understood.

The research there is (at least that which met our inclusion criteria) suggests IMGs may, for a number of reasons, work in occupations that do not necessarily match their skills or qualifications [45, 47]. If this is so, and if licensure examinations are a contributory factor, then in a world where physician shortages exist it seems appropriate to understand this better.

We have argued that the evidence for NLEs improving doctor performance and patient safety as a consequence of their introduction is weak, whereas the evidence for a correlation between test results and overall performance is strong. As such, the relative benefits of introducing a NLE may well be contingent upon the efficacy of existing regulatory systems. As such, policy-makers and regulators may consider moving beyond a one size fits all approach to NLE; evidence should be examined in light of existing regulatory systems and, as suggested above, the broader consequences of NLEs for issues such as workforce planning in relation to IMG doctors.

## Conclusions

The main conclusion of our review is that the debate on licensure examinations is characterized by strong opinions but is weak in terms of validity evidence. The validity evidence is particularly poor for the claim that NLEs improve, above and beyond existing assessments, either the competence of practitioners or the safety of patients. This is highlighted through mapping the evidence onto the APA framework: the evidence points to a relationship with other variables, including doctor performance and patient complaints, but there is not strong evidence that these outcome measures are a consequence of introducing NLEs. Nowhere, as yet, has staged a NLE to establish whether the introduction of such an examination significantly impacts upon measures of interest such as patient outcomes using a control group or a ‘control country’. While this raises significant political and practical challenges, in the absence of such evidence for these aspects of the NLEs, the debate looks set to continue.
